# Reg3γ: current understanding and future therapeutic opportunities in metabolic disease

**DOI:** 10.1038/s12276-023-01054-5

**Published:** 2023-08-01

**Authors:** Jae Hoon Shin, Nadejda Bozadjieva-Kramer, Randy J. Seeley

**Affiliations:** 1 Department of Surgery, University of Michigan, Ann Arbor, MI USA; 2 Veterans Affairs Ann Arbor Healthcare System, Research Service, Ann Arbor, MI USA

**Keywords:** Homeostasis, Type 2 diabetes

## Abstract

Regenerating family member gamma, Reg3γ (the mouse homolog of human REG3A), belonging to the antimicrobial peptides (AMPs), functions as a part of the host immune system to maintain spatial segregation between the gut bacteria and the host in the intestine via bactericidal activity. There is emerging evidence that gut manipulations such as bariatric surgery, dietary supplementation or drug treatment to produce metabolic benefits alter the gut microbiome. In addition to changes in a wide range of gut hormones, these gut manipulations also induce the expression of Reg3γ in the intestine. Studies over the past decades have revealed that Reg3γ not only plays a role in the gut lumen but can also contribute to host physiology through interaction with the gut microbiota. Herein, we discuss the current knowledge regarding the biology of Reg3γ, its role in various metabolic functions, and new opportunities for therapeutic strategies to treat metabolic disorders.

## Metabolic diseases: Role of antimicrobial peptide found in small intestine

An antimicrobial peptide, Reg3γ found in the small intestine may be a vital link between gut microbiota and metabolic regulation, and understanding its activity may inform future therapeutics. Reg3γ protects the host by keeping gut bacteria from passing into other organs. Questions remain regarding the role of Reg3γ in metabolic disorders. Randy Seeley at the University of Michigan, Ann Arbor, USA, and co-workers reviewed understanding of the biological roles of Reg3γ. Its production is boosted in infections as part of the host immune response, and it can confer beneficial effects in multiple organs. However, Reg3γ is downregulated in metabolic disorders such as obesity and type 2 diabetes. Stimulating the gut to generate high levels of Reg3γ can improve gut function, but also affect metabolic regulation in mice. Analogs designed to mimic Reg3γ may have therapeutic advantages.

## Introduction

Twenty-five years ago, the brain, muscle, adipose tissue, and pancreas were believed to be the key organs involved in regulating energy balance and glucose levels. However, current treatments for obesity and diabetes focus primarily on leveraging the GI tract to achieve sustained weight loss and improved glycemic control^1–3^. This strategy has increased the research attention given to the GI tract with the hope of identifying additional treatment strategies.

Much of this additional research attention has focused on the enteroendocrine cells that are known to produce hormones that can act on other organ systems. Our belief is that there are other cell types and gut functions that play equally important roles. For example, gut antimicrobial peptides have essential roles as a part of the innate immune response, which protects the host from external microorganisms^4–6^. One of these antimicrobial peptides, Reg3γ, is abundantly expressed throughout the small intestine, where it is an important component of the barrier that maintains spatial segregation of the gut bacteria from the host^7,8^. While the gut microbiota contributes to digestion and healthy gut function, it has also been hypothesized to be an important regulator of various aspects of energy homeostasis and glucose regulation^9^. However, the mechanism by which the microbiota influences host physiology remains contentious. Data obtained over the past few years highlight Reg3γ as a link between the gut microbiota and metabolic regulation. In this review, we describe key features of the physiological role and therapeutic potential of Reg3γ.

## Antimicrobial effects of Reg3γ

**Fig. 1:**
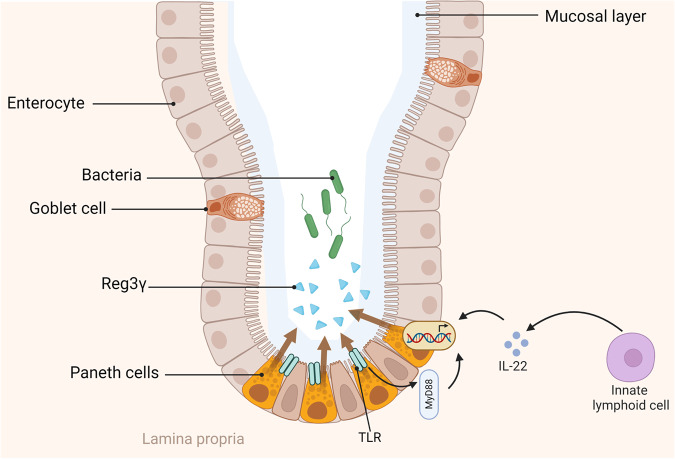
Regulatory mechanism of Reg3γ production. Reg3γ is mainly produced by Paneth cells, which directly respond to the gut bacteria through the Toll-like receptor (TLR)-Myd88 signaling pathway^22^. Reg3γ expression also induces IL-22 from innate lymphoid cells^23^. The figure was created with BioRender.com.

Reg3 proteins, as a part of the Reg family, were first isolated from rat regenerating islets in 1988^10,11^. This protein is also termed hepatocarcinoma-intestinal pancreas/pancreatitis-associated protein (HIP/PAP) because it was also identified in acute pancreatitis hepatocarcinoma^12,13^. The Reg3 family can be divided into four members termed Reg3α, Reg3β, Reg3δ and Reg3γ in mice, whereas humans have only REG3A and REG3G^14–16^. Members of the Reg3 family are abundantly expressed in the intestinal tract (Reg3α, Reg3β and Reg3γ) and pancreas (Reg3δ)^14,15^. Reg3γ containing an N-terminal prosegment maintains a biologically inactive state, whereas prosegment removal by trypsin or structural mutation enhances its antibacterial activity in the gut^17,18^. Reg3γ is mainly produced by Paneth cells and enterocytes in the small intestine, where it is secreted into the gut lumen (Fig. 1) and can exert bactericidal activity that preferentially targets gram-positive bacteria. Bacterial colonization is a key factor that triggers the production of Reg3γ in the gut under normal conditions^7,19^. Inhibition of bacterial colonization by antibiotic treatment or germ-free conditions suppresses the expression of Reg3γ, whereas supplementation with certain probiotics enhances Reg3γ expression^7,19–21^. Mechanistically, Paneth cells directly recognize and respond to bacterial signals through the TLR-MyD88-dependent signaling pathway^22^. Alternatively, IL-22 produced by innate lymphoid cells stimulates Reg3γ production^23^ (Fig. 1). It is important to note that Reg3γ does not impact the overall composition of the microbiome, but it affects mucus distribution and maintains the spatial separation of the gut bacteria from the intestinal epithelium^8,21^.

Furthermore, the production of Reg3γ can be altered by pathophysiological conditions. Studies have highlighted that bacterial infections cause a significant induction of Reg3γ, suggesting that Reg3γ has a protective role against infection^24,25^. Mucosal infection with *Listeria monocytogenes* and *Salmonella enteritidis* increases Reg3γ levels in the intestine, and Reg3γ is required to regulate mucosal inflammation in response to pathogenic bacterial infections^24,26^. Despite several lines of evidence indicating the causal role of Reg3γ in bactericidal activity as a part of host innate immunity, all of the data are not consistent. For instance, Reg3γ expression is increased in the urinary tract following uropathogenic *Escherichia coli* infection, while Reg3γ fails to kill pathogenic *E. coli*, and Reg3γ deficiency does not increase susceptibility^27^. These results suggest that Reg3γ production is induced not only by responses against microorganisms but also by directly recognizing bacterial products such as lipopolysaccharide or flagellin^19,28^.

Recent rodent studies from our laboratory and others reported that the expression of intestinal Reg3γ is downregulated in metabolic disorders induced by nutrition (high-fat diet, alcohol) or genetic modification (*ob/ob, db/db*) that result in obesity and impaired glucose regulation. Interestingly, multiple types of bariatric surgery result in increased intestinal expression of Reg3γ^21,29–31^. In addition, bile acids in the intestine and exogenous GLP-1 agonists in the pancreas stimulate Reg3γ production^32,33^. These data suggest that Reg3γ production is influenced by various metabolic conditions.

## Biological effect of Reg3γ

**Fig. 2:**
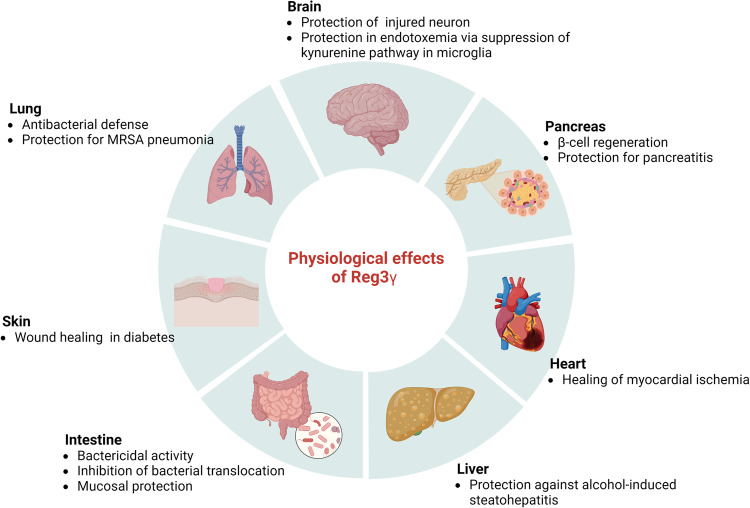
Pivotal role of Reg3γ as a host defense. Reg3γ acts on various organ systems by responding to damage or inflammatory disease. Abbreviation: MRSA, methicillin-resistant Staphylococcus. The figure was created with BioRender.com.

Studies in mouse models have suggested that Reg3 has beneficial effects in skin injury, such as psoriasis, colitis, pancreatitis, asthma, cardiac inflammation, alcoholic fatty liver, damaged brain neurons and graft-versus-host disease (GVHD) in allogeneic bone marrow transplantation^34–40^ (Fig. 2). The release of proinflammatory cytokines during the cutaneous inflammatory response stimulates antimicrobial peptides, including Reg3γ, which are critical for keratinocyte proliferation, differentiation and wound reepithelialization^38^. Reg3γ (or human REG3A) is abundantly expressed in skin lesions. In such situations, IL-17-induced IL-33 enhances Reg3γ expression in keratinocytes, in which Reg3γ inhibits TLR3-JNK2-induced inflammation via SHP-1^37^. In diabetic conditions, hyperglycemia reduces IL-33, which decreases Reg3γ and SHP-1 levels. This situation leads to increased TLR3 signaling, activation of JNK2, and impaired wound healing with more inflammation^37^.

During acute colitis in mice, IL-33 and Reg3γ are highly expressed in the colon^34,41^. IL-33 is produced by intestinal epithelial cells and promotes the production of Reg3γ in the gut during inflammation and mucosal recovery^42^. Studies in mice suggest that Reg3γ is a downstream mediator of IL-33 and has a protective role in colitis by reducing inflammation and oxidative stress^21,34,41^. Reg3γ also promotes anti-inflammatory responses via the JAK2/STAT3 signaling pathway, which can stimulate β-cell regeneration^36,43^. Treatment with an immunosuppressant drug, tacrolimus, inhibits STAT3-mediated transcript activation and causes β-cell failure, which is reversed by Reg3γ treatment by restoring insulin secretion and mitochondrial functions^44–46^. Reg3γ expression is elevated in a dose-dependent manner in caerulein-induced pancreatitis, and its deficiency exacerbates pancreatic inflammation^47,48^. However, in a murine model of chronic pancreatitis, Reg3γ promotes inflammation-associated pancreatic cancer progression^49^. These findings suggest a complex role of Reg3γ in inflammatory conditions.

While it is possible that many of these effects are a product of Reg3γ’s antimicrobial properties, there is strong evidence that Reg3γ can act to alter cellular signaling directly. Although Reg3γ is expressed at relatively low levels in the healthy pancreas^14^, its expression is elevated in diabetic islets in humans and mice^50,51^. Reg3γ overexpression in the pancreas via lentivirus injection promotes pancreatic β-cell regeneration, attenuates lymphocyte infiltration, and decreases the development of type 1 diabetes in NOD mice by activating the JAK2/STAT3 pathway^36^. Recently, exostosin-like 3 (EXTL3) has been identified as a binding protein for Reg3γ, and the Reg3γ-Extl3 signaling pathway has been implicated in regulating various cellular processes, including keratinocyte proliferation and differentiation, wound healing, and glucose homeostasis^37,38,52^. In addition, Extl3-deficient pancreatic β-cells have been shown to exhibit impaired glucose regulation and insulin secretion, along with abnormal islet morphology in mice^52^. These data suggest that Reg3γ provides metabolically beneficial effects that can act directly upon the endocrine pancreas to regulate glucose homeostasis. The exact mechanisms underlying the physiological and pharmacological actions of Reg3γ in metabolic tissues need further investigation.

## Gut manipulations for metabolic improvements and the role of Reg3γ

Bariatric surgery provides sustained weight loss and improved glucose metabolism in patients with obesity and/or type 2 diabetes (T2DM)^53,54^. Bariatric surgical procedures such as Roux-en-Y gastric bypass (RYGB) and vertical sleeve gastrectomy (VSG) manipulate the gut anatomy, which in turn forces the intestine to adapt to the new anatomy^55^. We and others have reported that RYGB and VSG in obese patients and rodents leads to increased levels of Reg3γ in the circulation and intestine^21,56,57^. VSG-operated WT mice lost weight and kept it off relative to their sham-operated controls. Reg3γ KO mice lost weight initially but regained weight such that they had the same weight and body fat as sham-operated controls^21^. Furthermore, the improved glucose homeostasis and decreased hepatic fat accumulation that occurs after VSG were not observed in mice lacking Reg3γ^21^. These data strongly suggest that Reg3γ is necessary for the beneficial effects of VSG. Nevertheless, Reg3γ’s primary role is not to respond to surgical alterations of the gut. A key question is what other manipulations of the gut alter Reg3γ to also benefit metabolic endpoints. Prebiotic treatment with oligofructose or inulin fiber reduces the deleterious effects of a high-fat diet to induce gut and metabolic dysfunction^58,59^. These dietary supplementations also restore Reg3γ production in the intestine^21,58,59^. Recently, we found that supplementation of a HFD with inulin fiber improves glucose tolerance relative to isocaloric cellulose fiber supplementation, while inulin’s ability to improve glucose tolerance is absent in Reg3γ-deficient mice^21^. The ability of both high-inulin-fiber diets and VSG to enhance gut barrier function is reduced in Reg3γ KO mice^21^. What do both bariatric surgery and high soluble fiber have in common? Notably, surgical and dietary interventions lead to an increased abundance of so-called “good bacteria” such as *Lactobacillus* and *Bifidobacterium* in the small intestine, and this happens in both WT and Reg3γ KO mice^21^. This outcome indicates that Reg3γ is not critical to the ability of these manipulations to induce changes in the composition of the gut microbiota. Are alterations in the microbiota driving changes in the increased Reg3γ expression? Specific bacteria such as *Bifidobacterium breve*, *Lactobacillus rhamnosus* GG, or probiotics containing multiple strains of *Lactobacillus* and *Bifidobacterium spp*. appear to increase Reg3γ expression in the intestine^20,21,60^. The protective effects of the good bacteria against gut barrier damage are reduced in mice that lack Reg3γ^21,61^. These data provide strong evidence that Reg3γ is required for these potent beneficial effects of the gut microbiota on host physiology in disparate gut manipulations by surgery, diet, or healthy options, such as VSG, inulin fiber diet or healthy bacteria intervention.

Recent studies have reported that the biological effects of metformin, a widely used treatment for T2D, arise from the intestine, which impacts key processes related to the glucoregulatory pathway^62,63^. Metformin not only induces Reg3γ expression but also enhances intestinal AMPK activity, which is required to mediate the therapeutic effect of metformin^62,64^. Zhang et al.^64^ showed that unlike WT DIO mice, metformin fails to restore intestinal Reg3γ expression in mice with intestine-specific deletion of AMPK. Given that metformin alters the gut microbiome by increasing the abundances of *Lactobacillus and Bifidobacterium spp*. in both humans and rodents^65–67^, these findings suggest that Reg3γ may modulate the glucose-lowering effects of metformin through the gut microbiota-AMPK-Reg3γ pathway.

## Therapeutic implications of Reg3γ

Our understanding of how different pathological conditions impact Reg3γ’s role in metabolism and gut function remains limited. For example, recent studies have found conflicting results on body weight and glucose metabolism in mouse models overexpressing or lacking Reg3 proteins in the gut^34,68–70^. Secq and colleagues reported that mice overexpressing PAP/HIP protein (REG3A) became obese with relatively high levels of glucose under normal nutritional conditions^68^. In contrast, Huang et al. found that Reg3γ overexpression in the gut protected mice from the negative effects of a high-fat diet, such as obesity and impaired glucose regulation. Additionally, increased Reg3 led to an increased abundance of *Lactobacillus* and expansion of macrophages that promote an anti-inflammatory response^69^. Another recent study, however, showed that neither Reg3γ deficiency nor intestinal overexpression affected diet-mediated obesity and glucose dysregulation^21,70^. As discussed above, studies using mouse models have suggested that enhancement of Reg3γ or Reg3γ-associated pathways might have a beneficial impact on metabolic homeostasis. Recently, studies have shown that the Reg3γ molecule could be leveraged for novel treatment strategies for type 2 diabetes^21,71,72^. Human REG3A administration through a transgene or recombinant protein improved glucose regulation in mice with obesity caused by high-fat diet or *ob/ob* mutation^72^. This effect was due to increased glucose uptake and decreased oxidative damage in skeletal muscle through activation of AMPK^72^. Moreover, the intramuscular expression of REG3A is negatively correlated with insulinemia, HOMA-IR, intramyocellular triglyceride and waist-hip ratio in healthy subjects^71^. Likewise, our finding that acute single injection of Reg3γ improves glucose tolerance in diet-induced obese mice supports the beneficial impact of Reg3γ^21^. The glucoregulatory action of Reg3γ can be mediated by interacting with Extl3 in the pancreas. Not surprisingly, Reg3γ-treated mice that lack Extl3 specifically in pancreatic β-cells displayed no improvement in glucose tolerance^21^. These data imply that Reg3γ can act via the circulation to exert beneficial effects.

While pharmacological application can be challenging without a clear understanding of efficacy, safety, and potential side effects, a promising approach is to modulate the composition of the gut microbiota that enhances Reg3γ activity by increasing the consumption of probiotics, prebiotics or fermentable fiber components that can confer beneficial effects to the host. Probiotic and prebiotic treatment prevents obesity, improves glucose homeostasis, and enhances gut function in both humans and rodents^73–77^. Intriguingly, *Lactobacillus* and *Bifidobacterium* species-containing probiotics and fermentable fiber-containing prebiotic supplementation not only stimulate Reg3γ production, but their beneficial effects also appear to be dependent on Reg3γ^21,30,78^. These observations lead to the question of how these bacteria exert their influence on Reg3γ. One possibility is that bacterially derived metabolites may increase Reg3γ. For example, propionate is a microbially produced metabolite that increases Reg3 lectin expression in cecal tissues and intestinal organoids. Propionate also ameliorates colitis in mice^79^. Interestingly, hepatic overexpression of REG3A has an effect inside the lumen to rescue the gut microbiota from oxidative stress and thereby attenuates inflammation in mice with colitis^34^. These recent reports imply that the protective effect of propionate on experimental colitis could be mediated through Reg3γ. Lactate and bile acids also have the potential to induce Reg3γ-related signaling as important mediators of metabolic function^21,32^. While further understanding of these pathways is needed, stimulation of endogenous Reg3γ in the gut may be a critical component for the treatment of metabolic diseases.

## Future perspectives

**Fig. 3:**
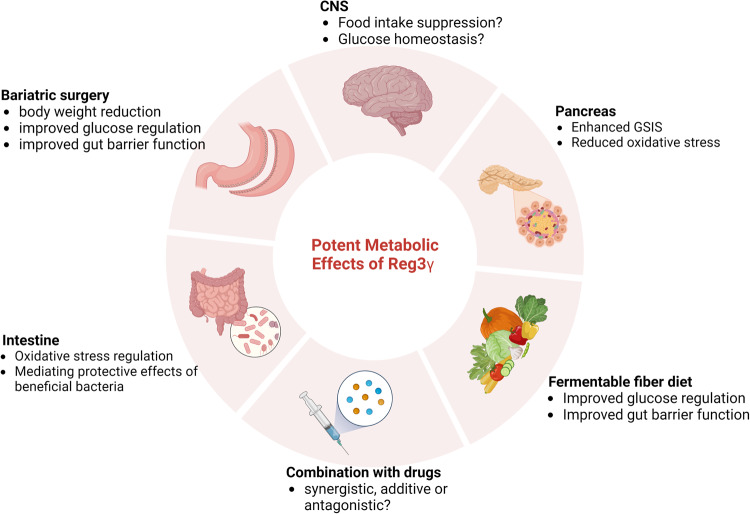
Potent effects of Reg3γ that regulate metabolic function. Reg3γ exerts metabolic benefits in various circumstances. Our analysis highlights the role of Reg3γ as a link between the gut microbiome and host physiology. Reg3γ is required for improvements in metabolic function after surgical or dietary interventions^21^. Reg3γ acts in the gut lumen to improve gut function (reduced oxidative stress, improved barrier function). Reg3γ enhances insulin secretion in the pancreas. Future studies are needed to examine whether Reg3γ plays a role in the central regulation of food intake and glucose regulation. Moreover, studies are needed to determine the therapeutic potency of Reg3γ. The figure was created with BioRender.com.

Reg3γ is abundant in the intestine under normal conditions, where it serves as an antimicrobial peptide that maintains the distance between the gut bacteria and the host, prevents bacterial translocation and regulates intestinal inflammation^8,26,80^. Reg3γ was also found to be a diagnostic and prognostic biomarker for predicting host responses to systemic inflammation^81–83^. In addition to animal studies, accumulating results indicate the clinical relevance of Reg3γ and related signaling pathways to metabolic disorders (Fig. 3). Reg3γ is secreted in the gut lumen and serves as a gut hormone that can act upon other organs in either an Extl3-dependent or -independent manner through its receptor. However, many questions about the tissue-specific actions of Reg3γ remain unanswered. For example, Reg3γ is produced in nociceptors in the dorsal root ganglion when exposed to LPS. It protects mice from LPS-induced endotoxemia by suppressing microglial IDO1 expression via the Extl3-Bcl10 axis^84^. Although the direct role of Reg3γ in the CNS has received relatively little attention, its receptor, EXTL3-positive cells, are widely distributed throughout the brain, including the cortical areas, hypothalamic nuclei, and brainstem^85^. Such data would indicate whether brain-penetrant analogs of Reg3γ might be useful therapeutically.

Finally, the possibility remains of combining Reg3γ/REG3A with other gut peptide-based therapeutics since strengthening the activity of Reg3γ/REG3A can also be directly or indirectly regulated by other therapeutic targets that have glucose-lowering ability. For example, Reg3γ expression is downregulated in the small intestine of Glp-1r-deficient mice^86^, thus implying that GLP-1 signaling may affect Reg3γ induction. Given that Reg3γ is likely to be degraded by DPP4^21^, it needs to be further determined whether the DPP4 inhibitor sitagliptin affects Reg3γ activity, which is associated with metabolic improvements. Further studies are needed to gain more molecular insights into its efficacy and potency, thereby identifying new potential targets.

In summary, Reg3γ, as a part of the host immune system, counteracts diverse stress factors, including oxidative stress and the inflammatory response. Reg3γ not only plays a role in the gut lumen but can also contribute to host physiology through interactions with the gut microbiota. The current state in the field of treating metabolic diseases points toward the effectiveness of manipulating the gut. While research into the role of Reg3γ in metabolic regulation is relatively new, emerging evidence indicates a potent role of Reg3γ in impacting metabolic function in other organs either via paracrine or endocrine action. Hence, appropriately designed analogs may provide unique therapeutic advantages by acting both within the gut and on other target organs. A key question for such analogs will be the degree to which they should replicate the antimicrobial actions or act as “Extl3 agonists” to exert beneficial actions. Finally, as we understand more about what drives endogenous Reg3γ, a variety of dietary supplements may be used to harness the beneficial effects of the endogenous Reg3γ system.
